# Quantifying Molecular-Level Cell Adhesion on Electroactive Conducting Polymers using Electrochemical-Single Cell Force Spectroscopy

**DOI:** 10.1038/srep13334

**Published:** 2015-09-03

**Authors:** Hongrui Zhang, Paul J. Molino, Gordon G. Wallace, Michael J. Higgins

**Affiliations:** 1 ARC Centre of Excellence for Electromaterials Science, Intelligent Polymer Research Institute, AIIM Facility, Innovation Campus, University of Wollongong, Wollongong, NSW 2522, Australia

## Abstract

Single Cell Force Spectroscopy was combined with Electrochemical-AFM to quantify the adhesion between live single cells and conducting polymers whilst simultaneously applying a voltage to electrically switch the polymer from oxidized to reduced states. The cell-conducting polymer adhesion represents the non-specific interaction between cell surface glycocalyx molecules and polymer groups such as sulfonate and dodecylbenzene groups, which rearrange their orientation during electrical switching. Single cell adhesion significantly increases as the polymer is switched from an oxidized to fully reduced state, indicating stronger cell binding to sulfonate groups as opposed to hydrophobic groups. This increase in single cell adhesion is concomitant with an increase in surface hydrophilicity and uptake of cell media, driven by cation movement, into the polymer film during electrochemical reduction. Binding forces between the glycocalyx and polymer surface are indicative of molecular-level interactions and during electrical stimulation there is a decrease in both the binding force and stiffness of the adhesive bonds. The study provides insight into the effects of electrochemical switching on cell adhesion at the cell-conducting polymer interface and is more broadly applicable to elucidating the binding of cell adhesion molecules in the presence of electrical fields and directly at electrode interfaces.

Electrically switchable surfaces are capable of on-demand, temporal control of cell adhesion. This is achieved by applying a voltage to an electrode, causing a switch of surface chemistry, to either promote or inhibit interactions with surface molecules present on the living cell surface. They are important for fundamental studies on cell interactions^1^, and increasingly used to manipulate cell adhesion for spatio-temporal detachment of cells^2^, electrochemical cell sensing^3^, electrophoretic cell trapping^4^, diagnostic protein arrays^5^, low-fouling biomaterials^6^, and envisaged as signaling platforms to enable electrical recording as well as novel delivery of physico-mechano-chemical signals to control the growth and development of cells in direct contact with the electrode^7^.

Strategies include the use of gold substrates functionalized with self-assembled monolayers consisting of charged end-groups or ligands. Upon electrical stimulation, these surface molecules are either electrochemically cleaved^8^, or electrostatically attracted toward or repelled from the electrode^9^, with the effect of shedding, hiding or exposing bioactive groups. Electrical control of cell adhesion is also achieved using conducting polymers with entrapped biomolecules that can switch their orientation, or freely diffuse, upon oxidation and reduction^9^,^10^. The general switching mechanisms involve controlling the presentation of surface ligands specific to cell surface receptors^11^, or bioactivity of cell recognition proteins adsorbed on the electrode surface^12^.

Most electrically switchable surfaces are capable of reversible and rapid switching. For this situation, it is predicted that cell adhesion will involve the cyclic breakage and formation of many individual bonds. Such rapid turnover of cell adhesion, as occurs in migration^13^, is an emerging mechanism in adhesion-mediated signaling pathways^14^ and increasingly implicated in cell processes such as regulation of stem cell populations^15^. Switching on or off the activation of receptors at specific time-points enables temporal regulation^15^. Endogenous or weak electrical fields and gradients in the cellular environment also polarize receptors and intracellular signalling molecules, altering their density and distribution, to control cell migration^16^. It is therefore conceivable that electrode surfaces could provide electrical control of physical bonds involved in cell signaling pathways.

An important question is how do the dynamic electrochemical signals of electrode surfaces affect cell adhesion at the molecular level? Studies to date on electrically switchable surfaces use optical imaging to monitor the effects on cell adhesion. These typically involve quantifying the amount of spreading or detachment of cells over a period of >30 minutes, which is incompatible with rapid switching and elucidating potential effects at the molecular level. One often needs to extrapolate whole cell morphological changes to possible dynamic, molecular processes of cell adhesion under electrical control. To address this, we need to directly probe in real-time the individual molecular bonds and force between the living cell surface and electrically switchable surface, which has hitherto been difficult to achieve. Here, we apply a technique termed Single Cell Force Spectroscopy (SCFS) to enable direct measurement of molecular force between a single cell and conducting polymer under electrical control.

## Results and Discussion

### Combining Electrochemistry and Single Cell Force Spectroscopy

**Figure 1:**
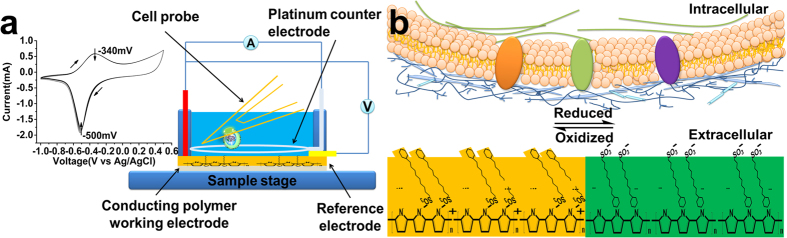
(**a**) Schematic of SCFS combined with a 3-electrode electrochemical cell mounted on a Bio-AFM. The electrochemical cell is temperature controlled at 37 °C, consisting of the PPy/DBSA substrate (orange) as the working electrode, a platinum wire ring (silvery grey) as the counter electrode, small Ag/AgCl reference electrode (yellow), and controlled by an external potentiostat. Cyclic voltammograms performed in CO_2_ independent medium indicate an oxidation peak at approximately −340 mV and reduction peak at −500 mV (left image). During the SCFS measurements, the adhesion force is detected between a single cell attached to the tip and the polymer substrate whilst applying a constant voltage. (**b**) Schematic showing an expanded view of the cell-conducting polymer interface. The hydrophilic and hydrophobic surface properties of the PPy/DBSA can be electrically switched via reorientation of the DBSA dopant. When oxidized (yellow substrate), negatively charged sulfonate group coordinate with the positively charged polymer chains. Upon reduction (green substrate), the DBSA switches orientation to enable favourable hydrophobic interactions between the dodecylbenzene groups and neutral polymer. Without the presence of proteins, the interaction occurs between the cell surface glycocalyx (blue) and dodecylbenzene or sulfonate groups of the polymer.

In this study, we combine the Single Cell Force Spectroscopy (SCFS)^17^,^18^ with Electrochemical-Atomic Force Microscopy, which is capable of dynamic, repeatable measurements of single cell adhesion on electrically switchable surfaces with force resolution down to 20 piconewtons on millisec to minute timescales. SCFS has made a significant impact by elucidating molecular mechanisms of integrin-extracellular matrix adhesion (e.g. collagen)^19^, including early stages of adhesion^20^, receptor cross-talk^21^ and effect of culturing agents^22^. Furthermore, the ability to repeatedly probe molecular interactions of the same cell on a material surface is important for gaining access to temporal and dynamic effects of electrically switchable surfaces. A detailed practical guide on SCFS and its advantages-disadvantages as a single cell measurement technique have recently been described^18^. We specifically probe the live L929 fibroblast single cell adhesion on polypyrrole (PPy) conducting polymer substrates doped with dodecylbenzene sulfonate (DBSA). PPy/DBSA functions as a working electrode within a 3-electrode electrochemical cell under the AFM probe (Fig. 1a). The setup allows cyclic voltammetry (Fig. 1a**, left)** and constant potential measurements whilst simultaneously measuring single cell adhesion on the working electrode. Cell viability before/after the measurements is confirmed using calcein staining (see methods section and Supplementary Figure S1).

To date most studies have investigated protein-mediated cell adhesion on conducting polymers, although the contribution from non-specific interactions is less clear. Non-specific interactions can override specific peptide-mediated cell adhesion^23^ and may also exert significant control over cell growth and development, representing an evolving strategy in the use of functional groups to enable “specific” interactions without using expensive peptides or bio-reagents^24^. Here, we use only serum-free media as the electrolyte to investigate intrinsic binding of cells to the conducting polymer substrate. Specifically, the incorporated DBSA with sulfonate groups is a potential mimic of sulfonated biopolymers such as glycosaminoglycans whose degree and patterning of sulfonation regulates binding with extracellular matrix proteins, cytokines and cell surfaces^25^.

**Figure 2:**
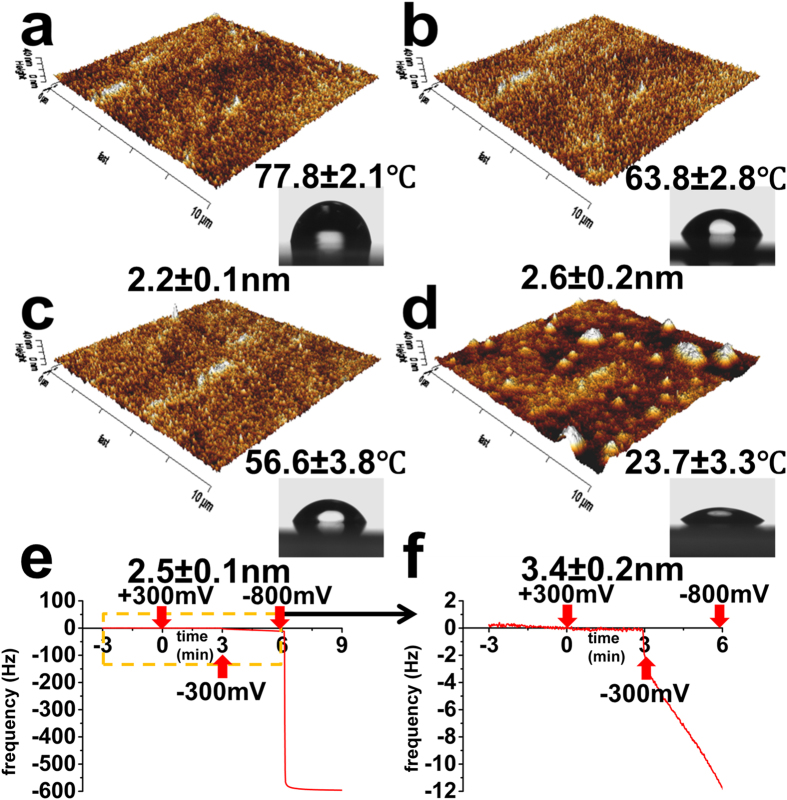
Three-dimensional AFM height images of the native PPy/DBSA polymer (**a**) and same film subjected to a series of applied voltages in the order of (**b**) +300 mV, (**c**) −300 mV and (**d**) −800 mV. Inset shows side-view optical images of milli-q water droplets on film under different surface potentials during goniometry measurements. Contact angle of the PPy/DBSA film at the different applied voltages are displayed above inset images. Values under images are surface roughness. (**e**) Frequency response from PPy/DBSA as a function of the series of applied voltages in accord with SCFS experiments. (**f**) Zoomed in region of dashed box in (**e**). Errors are standard error of the mean (s.e.m), n = 3 (n is number of measured polymer samples).

The electrochemical switching mechanism for PPy/DBSA involves rearrangement of the sulfonate and dodecylbenzene groups of the DBSA molecules within the conducting polymer (Fig. 1b). During oxidation, the negatively charged sulfonate groups coordinate with the positively charged polymer, causing the hydrophobic groups to orientate to the polymer-liquid interface^26^. The sulfonate groups and hydrophobic groups can then switch orientation during reduction, with the hydrophobic groups preferring to coordinate with the neutral polymer backbone. Changes in wettability from goniometry measurements during this process support this mechanism of switching^27^. During our SCFS measurements, we initially apply no voltage, followed by potentials of +300 mV, −300 mV, and then complete reduction of the PPy at −800 mV. This series of potentials gives decreasing substrate contact angles of 77.8 ± 2.1°, 63.8 ± 2.8°, 56.6 ± 3.8° and 23.7 ± 3.3° (mean ± s.e., n = 3), indicating a gradual increase in wettability, relevant to the extent of interfacial sulfonate groups, as the polymer becomes more reduced (Fig. 2a–d). *In-situ* electrochemical-AFM height images of the different polymer surfaces show that an applied voltage of −800 mV induces a change in surface morphology and ≈1 nm increase in r.m.s. surface roughness (Fig. 2a–d) that may be due to either uptake of electrolyte or structural rearrangement of PPy chains and/or DBSA. To further understand the process of electrolyte-ion uptake, QCM monitoring of frequency (*f*) and dissipation (*D*) was performed whilst applying a series of voltages with durations in accord with the SCFS measurements. Representative time-resolved QCM measurements revealing the *f* profiles in response to the series of applied voltages are shown in Fig. 2e–f. After equilibration, constant *f* signals are evident on the native polymer, which do not change during +300 mV, though small shifts in *f* (≈12 Hz decrease) occur during −300 mV. Notably, a further large decrease in *f*, or effective increase in mass representing almost half of the polymer film initial mass, occurs upon application of −800 mV, indicating a significant uptake of electrolyte/ions that is the likely cause for the changes in surface morphology and roughness during reduction of the polymer. The mass increase during reduction is indicative of cation movement into the polymer to charge-balance negatively charged sulfonate groups of entrapped DBSA.

### Molecular Interactions of Cell Adhesion on Conducting Polymers: Membrane Tethers and Cytoskeletal-linked Molecules

**Figure 3:**
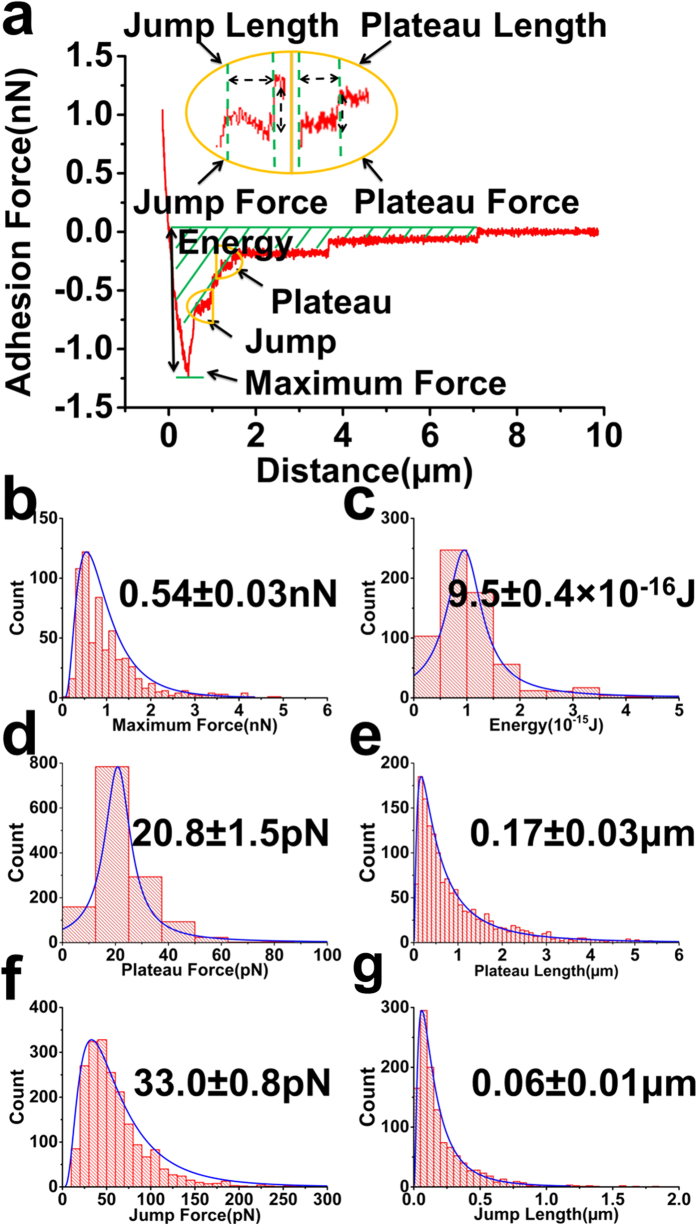
(**a**) Representative force-distance curves (retraction curve) of a single L929 cell adhesion on native PPy/DBSA polymer (no applied voltage). The peak force is given as the maximum force values required to detach the cell. Plateau and jump interactions are evident following the bulk detachment of the cell. (Inset) Plateau interactions show a constant force over a given distance, while jump interactions typically show a non-linear increase in force that is characteristic of an elastic response of a biological molecule under strain. Analysis of the force-distance curves including quantification of the maximum force, adhesion energy, and force/rupture length of the jump and plateau interactions. (**b**–**g**) Histograms of the (**b**) Maximum force (0.54±0.03nN); (**c)** Adhesion Energy (9.5 ± 0.4 × 10^−16 ^J); (**d**) Plataeu force (20.8 ± 1.5 pN); (**e**) Plateau length (0.17 ± 0.03 μm); (**f**) Jump force (33.0 ± 0.8pN); (**h**) Jump length (0.06±0.01 μm); (peak distribution ± s.e.m; nf = 650; nc = 22; )

Whilst applying a constant voltage, the cell is brought into contact with the conducting polymer with an applied force of 1 nN for a period of 1 sec, and then the adhesion force is quantified as the cell is retracted from the surface. For substrates with no applied voltage, referred to as native polymers, the force curves (FCs) show a profile that is typical of SCFS studies^17^,^18^,^19^ (Fig. 3a). A maximum cell adhesion force of 0.54 ± 0.03 nN (peak distribution value ± s.e.m; nf = 650; nc = 22; nf: number of analyzed FCs, nc: number of measured cells) (Fig. 3b) indicates the force required to detach most of the cell from the surface, followed by characteristic events involving plateaus and jumps (Fig. 3a). The integrated area under the entire interaction represents the adhesion energy (Fig. 3a**, green dashed)** and gives a value of 9.5 ± 0.4 × 10^−16 ^J (peak distribution value ± s.e.m; nf = 650; nc = 22) (Fig. 3c).

Plateaus are evident as a constant force of 20.8 ± 1.5 pN (peak distribution value ± s.e.m; nf = 650; nc = 22) (Fig. 3d) with a rupture length of 0.17 ± 0.03 μm (peak distribution value ± s.e.m; nf = 650; nc = 22) (Fig. 3e) and occur due to adhesion-induced formation of membrane tethers^28^. In this case, binding to cell surface molecules that have weak, or absent, physical linkage with internal cytoskeletal components results in dislocation of the binding complex, followed by extraction of lipids that form a tube, or membrane tether, as the cell moves away from the point of adhesion. The membrane tether force does not reflect the strength of the bond anchoring the molecules at its end to the polymer, but is described by lipid membrane properties such as bending rigidity and dimensions of the tether^29^. We measure a tether force of 20.8 ± 1.5 pN (peak distribution value ± s.e.m; nf = 650; nc = 22) (Fig. 3d), indicating interactions with individual tethers of <100 nm^30^ that are shown to have relevance as adhesion structures for neutrophil binding to platelets in thrombogenis^31^ or retrograde transport for communication between cells^32^.

Conversely, the jumps relate to unbinding of cell surface molecules that maintain their cytoskeletal linkages^18^, leading to stiffer bonds that break with force of 33.0 ± 0.8 pN (peak distribution value ± s.e.m; nf = 650; nc = 22) (Fig. 3f) at shorter rupture lengths of 0.06 ± 0.01 μm (peak distribution value ± s.e.m; nf = 650; nc = 22) (Fig. 3g). Without the use of extracellular matrix proteins, the maximum cell adhesion force, as well as jumps and plateaus, represent the very early events of cell attachment via the glycocalyx to the conducting polymer. SCFS on cells treated with hyaluroniase, an enzyme that degrades hyaluronic acid, show a reduced jump force and this is consistent with jumps being a result of interactions with major components of the glycocalyx^33^. The non-specificity of the glycocalyx-conducting polymer interaction means that identifying the specific molecule(s) involved is difficult to ascertain. Non-specific interactions are expected to comprise a mixture of interacting molecules, leading to variability in force and rupture lengths. However, previous SCFS on concanavalin functionalized substrates that bind various cell surface sugars show characteristic interactions for the glycocalyx^34^. Similarly for the jumps, we observe a force distribution with peak value at ≈33 pN (Fig. 3f) that corresponds to forces at the single molecule level. As the AFM analysis only considers isolated, individual jumps (see Supplementary Figure S2), the presence of a mono-modal force distribution (Fig. 3f), as opposed to a multiple peak distributions, further suggests the likelihood that single molecules are comparatively involved in the interactions.

### Effect of Electrical Switching on Single Cell Adhesion to Oxidized and Reduced Polymers

**Figure 4:**
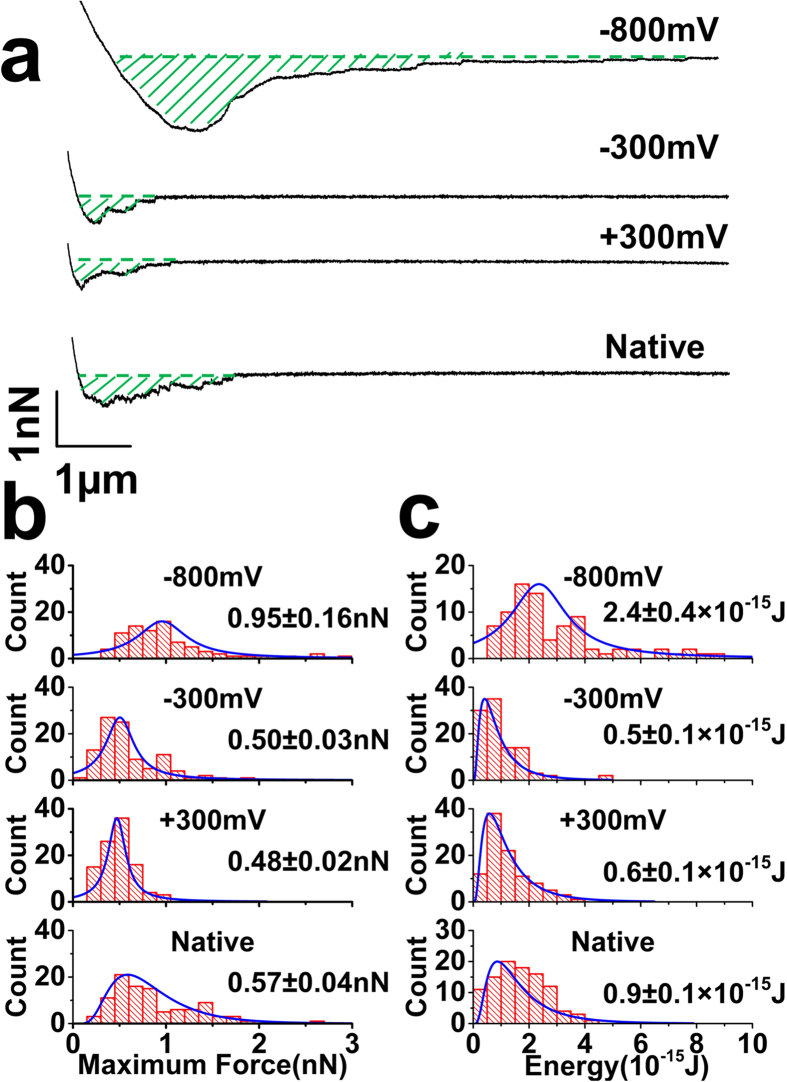
(**a**) Representative FCs for the different polymer surfaces. SCFS were taken in the order of native polymer, +300 mV, −300 mv and −800 mV. A greater maximum force and rupture length is observed for −800 mV. Comparison of histograms for the maximum force (**b**) and adhesion energy (**c**) of the native polymer, +300 mV, −300 mV and −800 mV. Both histograms for −800 mV show an increase in the peak distribution value. (Peak distribution ± s.e.m; nf = 91–100; nc = 10).

FCs initially taken on the native polymer exhibited similar maximum cell adhesion force to curves obtained using substrates exposed to +300 mV or −300 mV (Fig. 4a). Subsequent FCs obtained during exposure of the substrate to −800 mV show a ≈65–100% increase in the maximum cell adhesion force (Fig. 4a) with a value of 0.95 ± 0.16 nN (peak distribution ± s.e.m; nf = 91; nc = 10) (Fig. 4b) and accompanying significant increase in adhesion energy to 2.4 ± 0.4 × 10^−15 ^J (peak distribution ± s.e.m; nf = 91; nc = 10) (Fig. 4c). This effect of −800 mV in enhancing cell adhesion is evident by a shift in the peak distribution values of the maximum cell adhesion force (Fig. 4b) and energy (Fig. 4c) when compared to the native polymer, +300 mV, −300 mV, and shows statistical difference in ANOVA (F_critical _< F, p = 1.1e^−16 ^< 0.05) and post-hoc Tukey (q_critical _> q; p < 0.05). When considering the electrical switching mechanism in Fig. 1b, stronger adhesion on the reduced polymer indicates that sulfonate groups play a more dominant role in promoting cell adhesion via glycocalyx molecules compared to their contiguous hydrophobic, dodecylbenzene groups of the DBSA. This follows that under serum-free conditions, cells tend to adhere more on hydrophilic followed by hydrophobic substrates^13^. Concomitant with the increase in interfacial sulfonate groups, is the significant uptake of cations and associated solvent (CO_2_ independent and serum-free media) (Fig. 2e–f), which may also play a role in facilitating single cell adhesion. We also consider that a change in the stiffness or deformation of the cell modifies the effective interaction area between the cell and polymer surface, hence affecting the adhesion. To account for such changes, we fitted the contact region of the approaching curves to Hertz contact theory to quantify the Young’s modulus of cells during exposure to the series of applied voltages. A small increase in modulus at −800 mV (127 ± 8.9 Pa), compared to native polymers (112 ± 8.2 Pa), +300 mV (112 ± 8.0 Pa) and −300 mV (111 ± 7.7 Pa) (Supplementary Figure S3), would be expected to reduce the cell deformation under the same loading force and thus decrease the effective interaction area and cell adhesion. Therefore, the change in cell modulus is contrary to the increase in cell adhesion during electrochemical reduction at −800 mV.

**Figure 5:**
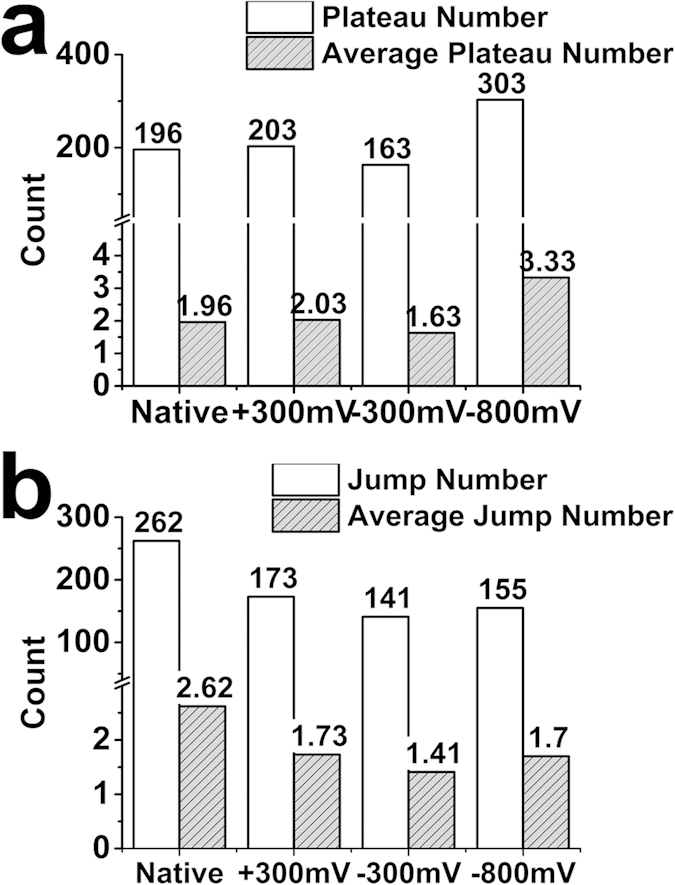
Statistical analysis of jump and plateau events. (**a**) Total number of observed plateau interactions obtained for each polymer surface (white bar). Total number is given above each bar. Average number of plateau interactions in each force curve performed on the different polymer surfaces (grey striped bar). The average number is given above each bar. (**b**) Same anaylsis for jump interactions. A higher number and average of plateau interactions occurs at −800 mV, while a higher number/average for jump interactions occurs on the native polymer.

**Figure 6:**
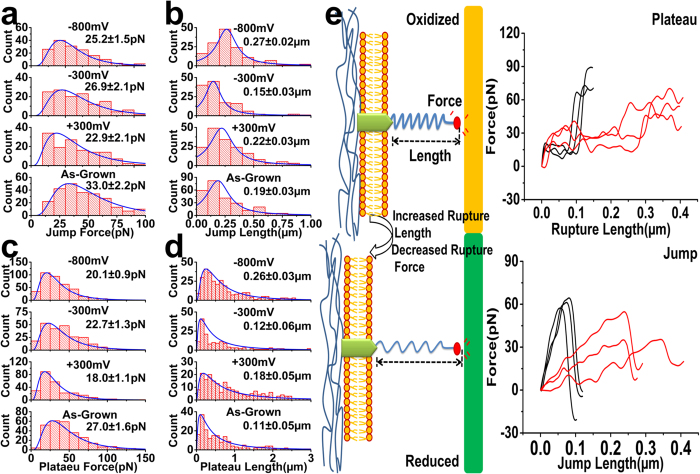
Histograms of jump force (**a**), jump length (**b**), plateau force (**c**) and plateau rupture length (**d**) for the different polymer surfaces. For both the plateau and jump force, the peak distribution value decreases as the polymer is switched from the native (oxidized) polymer to the +300 mV, −300 mV and −800 mV polymers. For both the plateau and jump rupture length, the final switch to −800 mv causes an increase in peak distribution values. (**e**) (Right images) Representative plateau and jump interactions obtained from FCs on native polymer (black curve) and reduced polymer (red curve). For the native polymer, the plateau force is higher and occurs over shorter lengths, while the jump force show a linear increase over short distances, resulting in a peak profile. In contrast, the reduced polymer shows longer plateaus with lower force, and jumps with a smaller linear increase in force over longer distances. These indicate that switching from the native polymer to reduced state causes a change in the positive gradient (pN/um) or stiffness of the jump interactions. (Left images) Schematic highlighting these changes in the glycocalyx-polymer bond properties, including the decrease in interaction force (**F**) and increase in rupture length (**L**) when switching from the native polymer to reduced state.

The total number of plateau interactions significantly increases at −800 mV (Fig. 5a), while the native polymer shows the opposite with a significantly higher number of jumps (Fig. 5b). Such a difference suggests plateaus and jumps may be ascribed to interactions with two different types of cell surface molecule(s), which preferentially bind to either the reduced (−800 mV) or oxidized (native) polymer during electrical switching. For instance, sulfonate groups on the reduced polymer are more likely to bind to those glycocalyx surface molecules associated with weaker, or absent, membrane linkages. Another partial explanation is that increased adhesion and subsequent extension of a cell as it is pulled away from the surface, could lead to higher tensile stresses, thereby weakening membrane–cytoskeleton linkages and giving rise to increased plateaus interactions on reduced polymers. Cells treated with cytoskeletal disrupting compounds show a higher probability of observing plateaus accompanied by a decrease in the plateau force^33^. We observe a decrease in the plateau force at −800 mV (20.1 ± 0.9 pN) (peak distribution ± s.e.m; nf = 91; nc = 10) compared to the native polymer (27.0 ± 1.6 pN) (peak distribution ± s.e.m; nf = 100; nc = 10) (Fig. 6c), suggesting a possible weakening of membrane-cytoskeletal linkages. However, applied voltages of +300 mV (18.0 ± 1.1 pN) (peak distribution ± s.e.m; nf = 100; nc = 10) and −300 mV (22.7 ± 1.3 pN) (peak distribution ± s.e.m; nf = 100; nc = 10) also show reduced plateau force (Fig. 6c), despite their lower maximum cell adhesion force (Fig. 4a,b) and lower number of plateaus (Fig. 5a), indicating that an increase in adhesion-induced tensile cell force does not necessarily correlate with lower plateau force. It is therefore not definitively clear that weakening of membrane-cytoskeletal linkages, i.e. through mechanical effects, is the cause of the increased membrane tether interactions for more adherent cells on reduced polymers. This aspect is discussed further below in relation to concomitant increases in rupture length of the plateau and jumps (Fig. 6b) at −800 mV, which again raises the possibility of structurally compromised membrane-cytoskeletal linkages.

In contrast, a greater number of jumps occur on native polymers (Fig. 5b) whose surface chemistry is determined by their initial electrochemical growth. These substrates are in the oxidized state but remain marginally hydrophilic with a contact angle of 77.8 ± 2.1°(mean ± s.e.m.; n = 3) (Fig. 2a), indicating that the DBSA does not completely orientate to present hydrophobic groups, as shown in Fig. 1b. Prior to electrical switching, conducting polymers typically show a pattern of doped and undoped regions^35^, reflecting nanoscale lateral variations in surface charge^36^ and electrostatic interaction force across the polymer^37^. Notwithstanding this heterogeneity in surface chemistry, the increase in jumps suggests that a type(s) of cytoskeletal-linked glycocalyx molecules may show a higher affinity for hydrophobic, dodecyl groups, or regions of different surface charge, which are more prevalent at the native polymer surface.

### Effect of Electrical Switching on Molecular-Level Interactions

Of significant interest is the effect of electrical switching on molecular-level forces and bonds of living cell surface molecules. In this regard, we observe an interesting phenomenon whereby the series of applied voltages through oxidized to reduced states has a similar effect on the force and rupture length of both plateaus and jumps (Fig. 6a–d). Firstly, the plateau force decreases by ≈15–25% after an electrical switch from the native polymer (27.0 ± 1.6 pN) (peak distribution ± s.e.m; nf = 100; nc = 10) to applied potentials of +300 mV (18.0 ± 1.1 pN) (peak distribution ± s.e.m; nf = 100; nc = 10), −300 mV (22.7 ± 1.3 pN) (peak distribution ± s.e.m; nf = 100; nc = 10) and −800 mV (20.1 ± 0.9 pN) (peak distribution ± s.e.m; nf = 91; nc = 10) (Fig. 6c). This effect shows statistical significance in ANOVA (F_critical _< F, p = 1.7e^−12 ^< 0.05) and post-hoc Tukey (q_critical _> q; p < 0.05). Furthermore, the final switch to −800 mV causes an ≈45–135% increase in plateau rupture length to values of 0.26 ± 0.03 μm (peak distribution ± s.e.m; nf = 91; nc = 10) (Fig. 6d), indicating an increase in the propensity of the membrane lipid reservoir to form tethers^38^ that again can be explained by weakening of membrane-cytoskeletal linkages. ANOVA (F_critical _< F, p = 2.2 e^−11 ^< 0.05) and post-hoc Tukey (q_critical _> q; p < 0.05) analysis confirms this significant increase in plateau length at −800 mV compared to the native polymer, +300 mV and −300 mV. Following a similar trend, the jump force decreases by ≈20−33% after an electrical switch from the native polymer (33.0 ± 2.2 pN) (peak distribution ± s.e.m; nf = 100; nc = 10) to applied voltages of +300 mV (22.9 ± 2.1 pN) (peak distribution ± s.e.m; nf = 100; nc = 10), −300 mV (26.9 ± 2.1 pN) (peak distribution ± s.e.m; nf = 100; nc = 10) and −800 mV (25.2 ± 1.5 pN) (peak distribution ± s.e.m; nf = 91; nc = 10) (Fig. 6a), with ANOVA (F_critical _< F, p = 8.8 e^−9 ^< 0.05) and post-hoc Tukey (q_critical _> q; p < 0.05) analysis confirming a significant difference. The switch to −800mV similarly causes an increase in jump rupture length by ≈80–170% with values of 0.27 ± 0.02 μm (peak distribution ± s.e.m; nf = 100; nc = 10) (Fig. 6b) and ANOVA (F_critical _< F, p = 1.9 e^−3 ^< 0.05) and post-hoc Tukey (q_critical _> q; p < 0.05) analysis showing statistical significance.

These findings indicate a trend that firstly involves a switch from the native polymer to the electrically stimulated polymers that causes a decrease in molecular-level binding forces associated with cell surface glycocalyx – PPy/DBSA interactions. This occurs despite a gradual decrease in polymer wettability during the series of applied voltages, suggesting that modifying the degree of sulfonate and/or dodecylbenzene surface groups does not affect the binding force, but rather it is a difference in surface redox characteristics specifically between the native polymer and electrically stimulated polymers, or the action of electrical charging, that appears to be responsible. Without identification of the groups involved in binding, it is difficult to know whether the change in force is due to the sampling of a different type of bond on the respective surfaces, i.e. native polymer versus +300 mV, −300 mV and −800 mV, or intramolecular changes within a single type of bond. For instance, electrical switching from the native polymer to either +300, −300 or −800 mV may modify the type of polymer or glycocalyx group(s) involved in binding and therefore magnitude of the plateau or jump force. It is alternatively conceivable that the electrode-electrolyte interfacial properties, such as the electrical double layer or pH, may affect the binding forces as the native polymer (with no applied voltage) is subject to electrical charging during the SCFS measurements. Following this decrease in force, the final switch to −800 mV also results in lengthening of the plateau and jump interactions. Rudimentarily, this implies a decrease in the stiffness of linkage or structural elements, such as the cytoskeletal membrane components, glycocalyx molecules and polymer surface groups, which effectively act as spring on the bond. Figure 6e provides force profiles of individual plateau and jumps, as well as an accompanying schematic of the cell glycocalyx-polymer bond to highlight the changes in the interaction length (and force). Again, this may be related to surface chemistry that is specific to the reduced polymer, electrolyte-uptake induced swelling, or due to direct electrical effects such as electrical field gradient or electrical double layer as the polymer is constantly charged at −800 mV. In particular, a significant uptake of electrolyte and associated swelling is likely to cause changes in the mechanical properties of polymer. An expansion of the polymer and increase in hydration may ‘liberate’ polymer interacting groups, increasing their flexibility or enabling them to more freely interact. Ensuing changes in their mechanical properties may underlie a change in the stiffness of the molecular interactions. Alternatively, application of 2 V/cm DC current electrical fields is shown to cause a twofold decrease in cell elasticity and depletion of intracellular ATP^39^. Reduced ATP leads to inhibition of linker proteins that physically couple the cell membrane to the cytoskeleton. Separation of the membrane from the cytoskeleton is seen as a twofold increase in tether length extracted from the cell after electrical stimulation. Although the exact mechanism for a decrease in bond stiffness is unclear, it is interesting to consider that we may be observing a similar electrical effect on cell elasticity that manifests at the molecular level of glycocalyx-conducting polymer interactions.

In conclusion, the strength of initial events related to cell adhesion increase on DBSA doped conducting polymers as they are electrically switched from oxidized to reduced states. As the polymer is reduced, an increase in surface sulfonation, hydrophilicity and significant electrolyte uptake and polymer swelling correlates with the increase in single cell adhesion. Without the presence of proteins, the non-specific adhesion occurs primarily between the cell surface glycocalyx and sulfonate/dodecyl benzene surface groups of the polymer. At the molecular level, the glycocalyx interactions involve molecules that have either a weak, absent linkage to the intracellular cytoskeleton, resulting in interactions with membrane tethers, or those that have stronger cytoskeletal-linkages. For the latter, binding forces of ≈33 pN with a mono-modal distribution suggest the sampling of interactions at the single molecule level. Electrical switching modifies the molecular bond properties, including both the force and stiffness, of the glycocalyx-polymer interactions. This may be due to switching of specific surface chemical groups, electrolyte uptake and/or direct electrical effects at the electrode-electrolyte interface. This work provides a platform to enable insight into the effect of electrical switching and stimulation, as well as changes in redox properties, on molecular-level interactions between living cells and conducting polymers; it will more broadly be applicable to elucidating the bond properties and kinetics of specific cell adhesion molecules such as integrins in the presence of electrical fields and directly at electrode interfaces.

## Methods

### Electrochemical Polymerization of Polymer Films

The pyrrole monomer was purchased from Merck and purified by distillation over molecular sieves prior to use under nitrogen. The chemical dopant was sodium dodecylbenzenesulfonate (DBSA) (Sigma-Aldrich, 289957). Aqueous solution for electrochemical polymerization consisted of 0.2 M pyrrole in deionised milli-q water (18.2 MΩ) with 2 mg ml^−1^ DBSA. Gold coated Mylar was prepared by cutting 2.2 × 2.2 cm square pieces. Polypyrrole substrates doped with DBSA (PPy/DBSA) were grown galvanostatically under constant current (0.10 mA/cm^2^) for 10 min using an eDAQ EA161 potentiostat and recorder. The polymer growth was specifically performed in a JPK Electrochemistry Cell (ECCell™) with the gold coated Mylar as the working electrode (active electrode area of 2.0 cm^2^), a platinum wire counter electrode and Ag/AgCl reference electrode (DRIREF-2SH, World Precision Instruments). After growth, the films were washed with Milli-Q water, gently dried with N_2_ gas and placed in a drying oven until use.

### Contact Angle Measurements

A goniometer (KSV instruments Ltd.) was used to measure the contact angle of a freshly grown, native polymer substrate without an applied potential. After measurements on the native polymer, the same film was dried under N_2_ gas, placed in the JPK Electrochemistry Cell with CO_2_ independent medium (18045-088, Life Technology) and a constant potential of +300 mV was then applied to the film for 5 minutes using a eDAQ EA161 potentiostat. The film was then rinsed with milli-q water (18.2 MΩ), gently dried under N_2_ gas and goniometry immediately carried out on the film to determine the contact angle. This process was repeated on the same film for potentials of −300 mV followed by −800 mV. Measurements were repeated using 3 different polymer film samples.

### Quartz Crystal Microbalance

Electrochemical polymerization of PPy/DBSA films was performed using a Q-Sense electrochemistry module (QEM 401) axial flow cell with a Q-Sense E4 quartz crystal microbalance system (Q-Sense AB, Västra, Frölunda, Sweden), as described previously^40^. The QCM sensor was an A–T cut quartz crystal with a 10 mm diameter gold electrode (QSX301) with a fundamental resonance frequency of 5 MHz (Q-Sense AB, Västra, Frölunda, Sweden). Aqueous solutions for electrochemical polymerization were the same as above, consisting of 0.2 M pyrrole in deionised milli-q water (18.2 MΩ) with 2 mg ml^−1^ DBSA. The Q-Sense electrochemistry cell consisted of a platinum counter electrode, a World Precision Instruments Dri-ref reference electrode, and gold working electrode upon the quartz sensor. PPy/DBSA films were grown galvanostatically onto gold-coated Q-Sense quartz sensors using an eDAQ e-corder 410 recorder and EA163 potentiostat connected to the Q-Sense electrochemistry module. Aqueous polymer growing solution was flowed through the electrochemistry module at 10 μL/min, and films were grown at a current density of 0.1 mA/cm^2^ for 10 min. The quartz sensors were then removed from the E-cell and rinsed in distilled water and dried under a flow of nitrogen gas.

The PPy/DBSA coated sensors were transferred to back into the standard QEM-401 Q-Sense electrochemical cell as described above, with a three-electrode electrochemical cell setup employed with the polymer upon the Q-Sense sensor acting as the working electrode and equilibrated in CO_2_ independent media for 60 min at a constant temperature of 22 ± 0.02 °C.

Using EChart v. 5.5.18 (eDAQ) data acquisition and analysis software, a series of applied voltages was applied to polymer film whilst monitoring the QCM frequency (*f*) and dissipation (*D*) signals. *f* and *D* were initially measured for the native (as-grown, oxidized) polymers followed by the polymer with constant applied potentials of +300 mV, −300 mV and −800 mV. Experiments were repeated on 2 sample films.

### AFM Imaging

AFM imaging of the PPy/DBSA films on native polymers and whilst applying potentials of +300 mV, −300 mV and −800 mV was performed using the JPK Biowizard II with electrochemistry cell in CO_2_ independent cell medium (18045-088, Life Technology). Images were acquired in contact mode using cantilevers with spring constant of ~0.12 N/m (NP-O10, Bruker). Images were first collected on native polymer with no applied potential. The tip was then retracted and a constant potential of +300 mV was applied using an eDAQ EA161 potentiostat. Imaging was resumed after the current had reached steady-state. This process was repeated for applied potentials of −300 mV and −800 mV. Scans of 10 μm were performed at a scan rate of 1 Hz. Experiments were repeated on 3 sample films.

### AFM Probe Functionalization

AFM tipless probes (NP-O10 from Bruker) were firstly calibrated for their spring constant using the thermal method^41^ and then plasma cleaned for 20 mins. The probes were incubated in 0.5 mg/ml biotin-BSA (bovine serum albumin, biotinamidocaproyl-labeled) (A6043, Sigma) for 12 hours at 4 °C. After rinsing with PBS (P5368, Sigma), the probes were incubated in 0.5 mg/ml streptavidin solution for 1 hour at room temperature, followed by further rinsing with PBS. To enable covalent coupling of concanavalin-A, the probes were finally incubated in biotin-concanavalin-A (C2272, Sigma) for 1 hr at room temperature and rinsed with PBS. After functionalization, the probes could be stored at 4 °C for up to two weeks.

### Single Cell Force Spectroscopy (SCFS)

SCFS was performed using a JPK Biowizard II mounted on a fully automated inverted Nikon microscope, with the 3-electrode electrochemistry cell integrated onto the AFM sample stage. The instrument was enclosed in a cell incubation system for temperature and humidity control. The electrochemical cell also enabled local temperature control of the sample and consisted of a freshly grown PPy/DBSA polymer film as the working electrode, platinum wire counter electrode and small Ag/AgCl reference electrode. Electrochemical voltage and current signals/recording were controlled via an Edaq potentiostat and recorder (eDAQ EA161). For the SCFS, L929 cells were resuspended in 1 ml CO_2_ independent cell culture media (18045-088, Life Technology) at a density of 1 × 10^4 ^~ 5 × 10^4 ^cell/mL and injected into the electrochemical cell, which was maintained at 37 °C. Cells were allowed to settle onto the polymer film for only 5–10 mins to ensure they did not spread and adhere to the surface.

A functionalized AFM probe was lowered toward the surface, and prior to attaching a cell, a force curve was performed to measure the sensitivity. The probe was positioned over a cell and contact was made with a force of 1 nN for 5 secs followed by retraction of the probe with attached cell. Visualization of both the cantilever and cell with the inverted microscope and control of the cell sample by a motorized stage with step resolution of ≈<0.5 microns enabled precise positioning of a single cell at the end of the AFM cantilever. The cell was allowed to establish adhesion for 5–10 minutes on the Concanavalin A functionalized cantilever prior to the SCFS measurements, an important procedure for ensuring that cell adhesion to the cantilever is greater than adhesion to the opposing surface^42^. The live cell probe was then repositioned over the polymer to collect 10 FCs and SCFS firstly performed on the native polymer with no applied potential. The live cell probe was then retracted for 50 μm and a constant voltage was applied. FCs measurements resumed once the current had reached steady-state (~30 secs) and were performed during the electrical stimulation. This procedure was performed on the same live probe for the native polymer followed by applied potentials of +300 mV, −300 mV and −800 mV. 10 FCs were collected on each of the different surfaces. A waiting time of 10 sec was used between each FC with the probe retracted ~10 μm from the surface. FCs parameters included a loading force of 0.5 nN, dwell time of 1 sec, force retraction distance of 10 μm and retraction speed of 5 μm/s. A total of 52 cells were measured. 22 cells were measured on native polymers without electrical stimulation. In electrical stimulation experiments, the series of measurements on the native polymer followed by +300 mV, −300 mV and −800 mV, were obtained with 10 cells.

### Force Curve Analysis and Statistics

Analysis of the AFM images and FCs was done in the JPK Data Processing software (Version spm-5.1.4), which enabled automated detection and processing of jumps and plateaus. Following this, only those isolated, individual jumps and plateaus not convoluted by other interactions were considered for analysis of the forces and rupture lengths. Histograms of the force distributions were plotted and primarily fitted to either Lorenzian or Lognormal functions using OriginPro 9.1 to extract the peak distribution values. Histograms were prepared using the same bin size and peak distribution values obtained from the fitting. ANOVA and post-hoc Tukey were performed using statistical packages of OriginPro 9.1 and Igor Pro (Wavemetrics). To quantify the cell modulus, we fitted the contact region of the approaching curves to the Hertz model using the JPK Data Processing software.

### Cell Culture

Mouse fibroblast L929 cell lines were originally sourced from ATCC (CCL-1TM). L929 cells were cultured in Dulbecco’s Modified Eagle’s Medium (DMEM) (12800017, Life Technology) supplemented with 10% Fetal Bovine Serum (FBS) (10099141, Life Technology) and 3.7 g/L NaHCO_3_ (S5761, Sigma). Cells were cultured at 37 °C in a humidified, 5% CO_2_ atmosphere (HERA cell 150, Thermo) and were subcultured every two days for the experiments.

### Calcein Staining of L929 Cells

The live cell stain was stored as 1 mM Calcein AM (C3100MP, Life Technology) in DMSO(C-3099, Life Technology). For live staining of cells, a dilute 2 μM (1:500) solution of Calcein AM was prepared in PBS. The calcein AM solution replaced the culture media and live cells were incubated for 15 minutes at 37 °C. Cells were then removed from the calcein solution and rinsed 3 times in fresh PBS. Cell were then resuspended in CO_2_ independent media 1 ml CO_2_ independent cell culture media (18045-088, Life Technology) at a density of 1 × 10^4 ^~ 5 × 10^4 ^cell/mL and injected into the electrochemical cell of the AFM. Live cells AFM probes for SCFS measurements were then prepared (as described above) and a series of FCs during electrical stimulation were obtained. To confirm cell viability, fluorescent images were taken of the live single cell probe before (Fig. S1a) and after (Fig. S1b) measurements with electrical stimulation. Images were also collected from cells settled on the working electrode for 30 mins without electrical stimulation to assess general cell viability in the electrochemical cell (Fig. S1c).

## Additional Information

**How to cite this article**: Zhang, H. *et al*. Quantifying Molecular-Level Cell Adhesion on Electroactive Conducting Polymers using Electrochemical-Single Cell Force Spectroscopy. *Sci. Rep*. **5**, 13334; doi: 10.1038/srep13334 (2015).

## Supplementary Material

Supplementary Information
