# Supplementary material for: Quantifying Molecular-Level Cell Adhesion on Electroactive Conducting Polymers using Electrochemical-Single Cell Force Spectroscopy
Source: Sci Rep. 2015 Sep 3;5:13334. doi: 10.1038/srep13334 (PMC4558606; doi:10.1038/srep13334)
Supplement: Supplementary Information [file srep13334-s1.pdf]

SUPPLEMENTARY INFORMATION

Quantifying Molecular-Level Cell Adhesion on Electroactive Conducting Polymers using Electrochemical-Single Cell Force Spectroscopy

Hongrui Zhang[1], *Paul J. Molino*[1], *Gordon G. Wallace* [1], and *Michael J. Higgins* [1*]

[1]ARC Centre of Excellence for Electromaterials Science, Intelligent Polymer Research Institute, AIIM Facility, Innovation Campus, University of Wollongong,

Wollongong, NSW 2522, Australia.

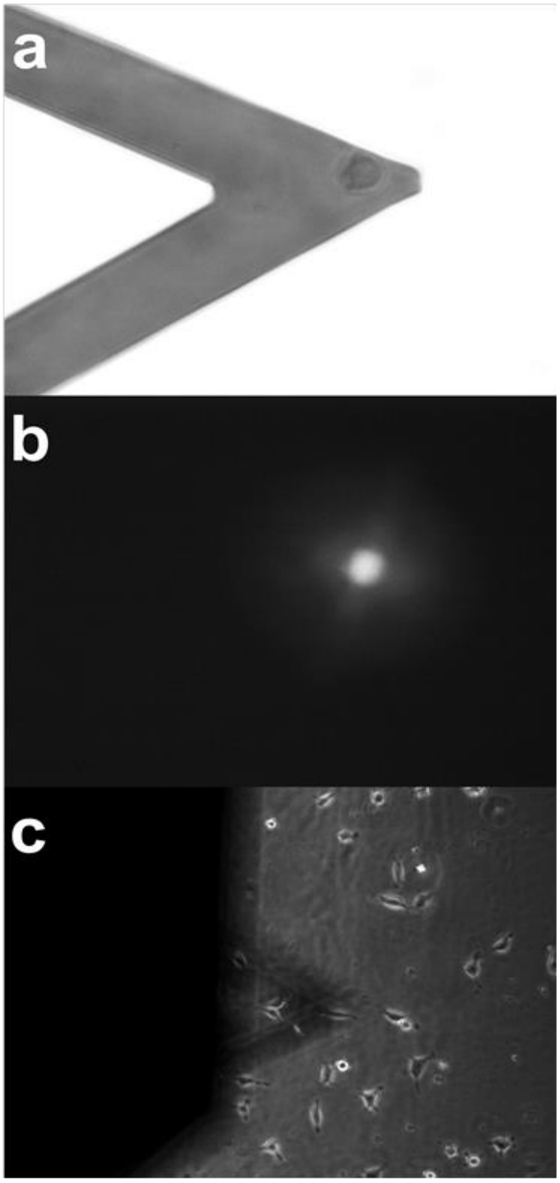

Figure S1. **(a)** Optical x40 microscope image of live calcein stained cell picked up via a tipless functionalized probe. **(b)** Corresponding fluorescent image calcein stained cell after a total of 40 FCs were taken on the native polymer and also whilst applying the series of applied voltages, +300 mv, -300 mv and -800 mv. These measurements to confirm the viability of cells after electrical stimulation was repeated with 3 live cell AFM probes. **(c)** Optical microscope image of L929 cells on PPy/DBSA substrate after injecting a cell suspension and allowing cells to settle for 30 minutes. Most cells spread and rapidly developed lamellipodia, indicating viable cells within the CO2 independent media and temperature-controlled electrochemical cell.

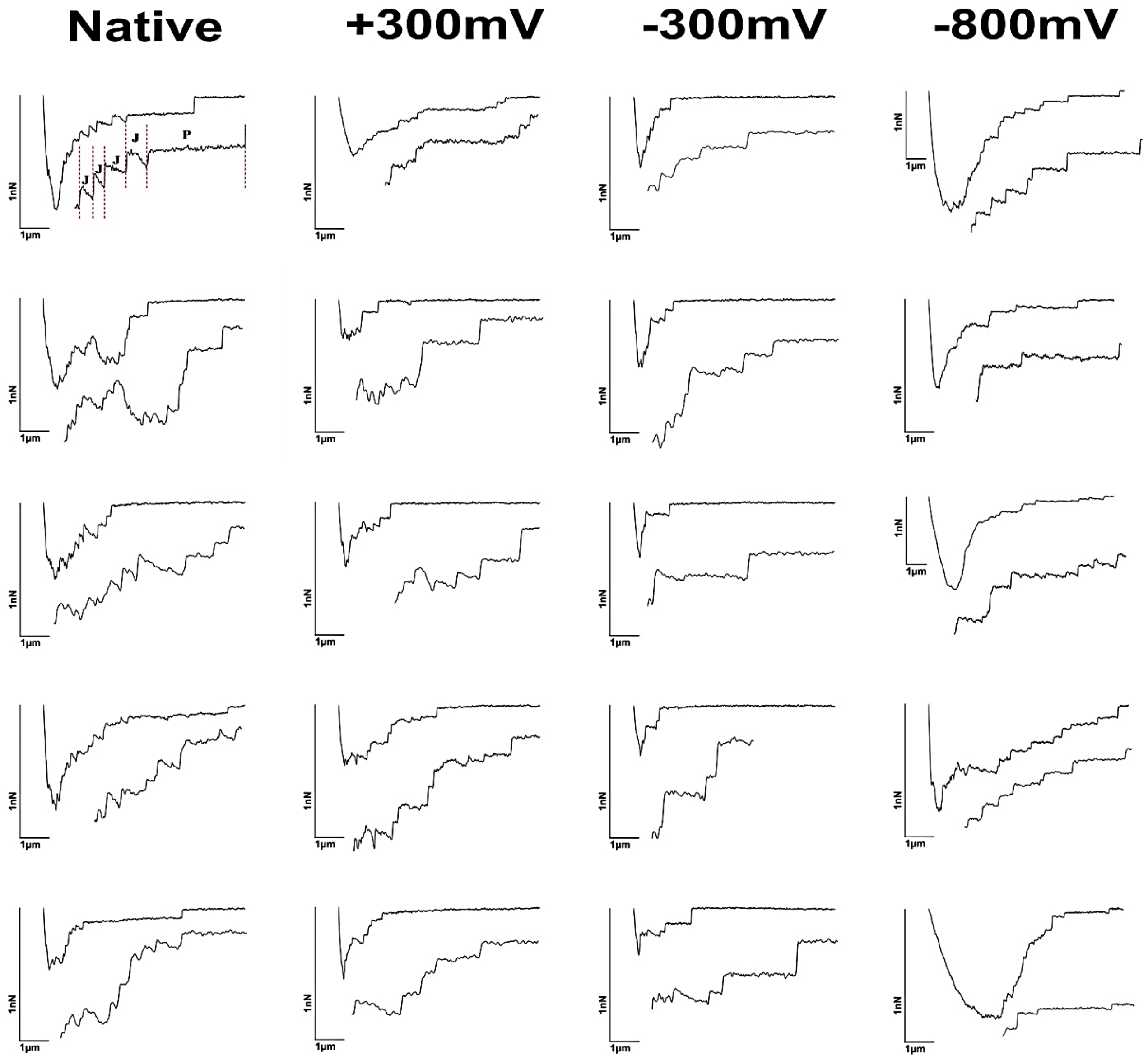

Figure S2. Force curves of single cell L929 adhesion on PPy/DBSA in CO_2 independent cell culture media at 37 degrees. Force curves are for the native polymer (no applied voltage), +300 mV, -300 mV and -800 mV. In each the top curves show the entire force versus distance curve with corresponding scale bars. The curves below show an expanded region of the curve to highlight the jump and plateau interactions. The top left curve (native polymer) also includes dashed lines to delineate the jumps (J) and plateaus (P). Only jumps and plateaus showing a clear single peak or step, respectively, were used for analysis of the force and rupture lengths.

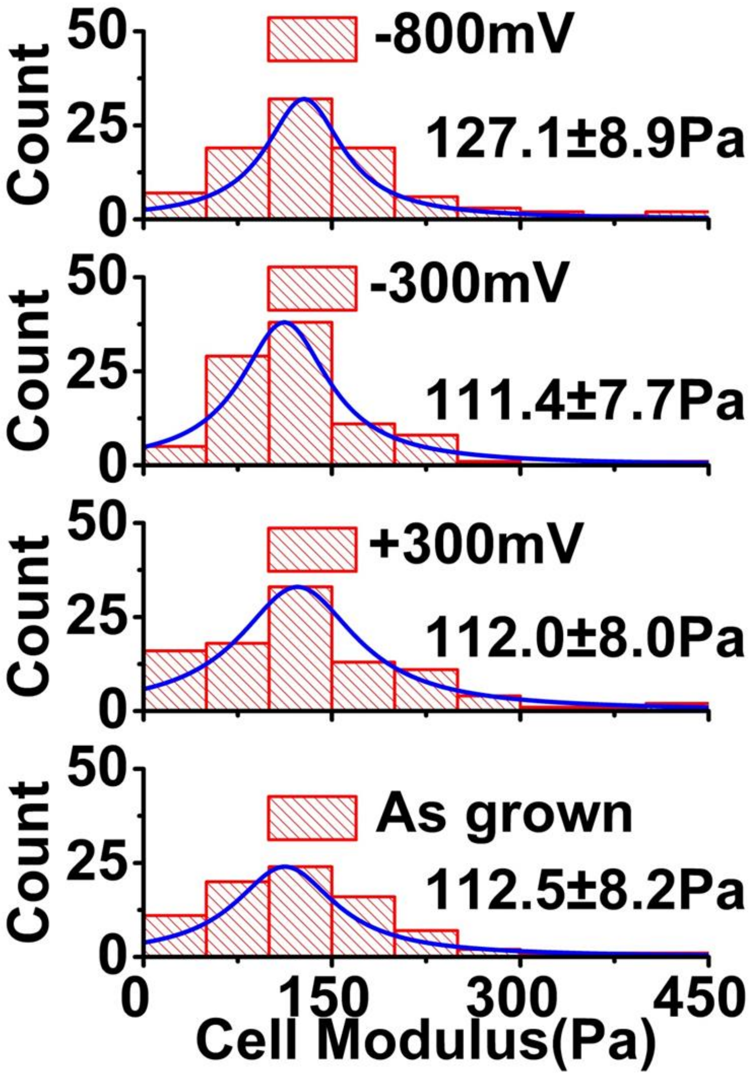

Figure S3. Histograms of cell modulus as a function of the applied voltage. (Peak distribution ± s.e.m; nf=91-100; nc=10). To quantify the cell modulus, we fitted the contact region of the approaching curves to the Hertz model using the JPK Data Processing software (Version spm-5.1.4).
